# Roles of histone lysine methylation in neurodevelopment and related disorders

**DOI:** 10.1002/ibra.70022

**Published:** 2026-06-04

**Authors:** Yun Chen, Yi Zhang, Hong Zhang, Xiaoli Liang

**Affiliations:** ^1^ School of Anesthesiology Zunyi Medical University Zunyi China; ^2^ Department of Anesthesiology the Second Affiliated Hospital of Zunyi Medical University Zunyi China; ^3^ Department of Anesthesiology the Affiliated Hospital of Zunyi Medical University Zunyi China

**Keywords:** Epigenetics, H3K4me3, histone modification, lysine methylation, neurodevelopmental disorders

## Abstract

Neurodevelopment is a highly ordered, precisely regulated process that establishes the neurobiological foundations of cognition, thought, emotion, and behavior. Neurodevelopmental disorders (NDDs) display marked phenotypic and genetic heterogeneity and variably impair learning, daily functioning, and social adaptation. As a key epigenetic mechanism, histone lysine methylation shapes chromatin accessibility and transcriptional programs, exerting central roles in neural stem cell fate decisions, neuronal migration and circuit assembly, as well as synaptic plasticity and learning and memory. Focusing on the activating histone H3 lysine 4 (H3K4) methylation marks (H3K4me1/2/3), this review synthesizes evidence for the bidirectional regulation mediated by “writers” (the KMT2/SET/DOT1) and “erasers” (the KDM/LSD and KDM5) in NDDs, including Kabuki syndrome, Wiedemann‐Steiner syndrome, autism spectrum disorder, and schizophrenia. We further outline how animal models, patient‐derived brain organoids, and multi‐omics atlases enhance mechanistic insight, and we discuss the translational potential of small‐molecule interventions, and metabolic modulation. Together, we summarize how the precise balance of methylation writing and erasure—and its crosstalk with DNA methylation and histone acetylation—forms an epigenetic network that drives neurodevelopmental programs. Targeting this network offers testable therapeutic avenues for NDDs.

## Graphical abstract

This graphical abstract centers on the theme of dynamic balance and systematically outlines the role of histone lysine methylation in neurodevelopment and related disorders. The figure first presents two key classes of regulators that control this balance—“writers” and “erasers”—and then indicates how interactions among different epigenetic modifications collectively regulate neurodevelopmental processes. On this basis, the figure further associates H3K4me3 with diseases and discusses therapeutic strategies as well as emerging technologies in research. The figure summarizes a complete logical chain from molecular regulation to developmental processes, and further to disease mechanisms and translational applications.

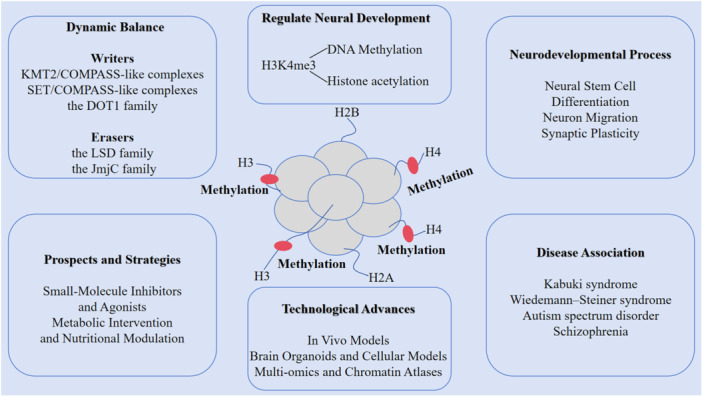

## 1 INTRODUCTION

Neurodevelopment refers to the dynamic process by which the human nervous system gradually matures, refines, and becomes more complex in structure and function from embryonic formation to early adulthood; it is not merely about the brain getting larger, but rather an extremely precise “construction project” guided by the genetic blueprint and profoundly shaped by experience and environment. Neurodevelopmental disorders (NDDs) are a group of chronic, developmental brain dysfunctions caused by genetic or acquired etiologies. Clinically, they are characterized primarily by impairments in cognition, social interaction, communication, motor behavior, or development, and they exhibit pronounced phenotypic and genetic heterogeneity.^1^, ^2^ The causes of NDDs are complex and include environmental and genetic factors. Although these disorders are often considered to arise in infancy or childhood, they may persist throughout life, and NDDs are diagnosed more frequently in males than in females.^3^ Epigenetics investigates mechanisms by which heritable changes in gene activity occur without alterations to the underlying DNA sequence, chiefly encompassing DNA methylation, histone modifications, and non‐coding RNAs.^4^ DNA methylation refers to the addition of a methyl group at specific sites on the DNA molecule, typically on cytosine bases.^5^ DNA is wrapped around histones to form chromatin; histone modifications occur mainly on the N‐terminal tails, where various chemical groups (e.g., acetyl, methyl, and phosphate) can be added or removed.^6^ Non‐coding RNAs are RNA molecules that do not encode proteins but play important roles in gene expression and regulation.^7^ Histone modifications play key roles in neurodevelopment, owing to the extraordinary complexity of brain development. This process entails the precise production, migration, and differentiation of hundreds of millions of neurons and the formation of functional synaptic connections, all governed by highly intricate and spatiotemporally specific gene‐regulatory networks. Histone modifications—particularly lysine methylation—provide the core molecular basis for achieving such fine‐tuned regulation.^8^ Accordingly, brain development depends on precise control of specific genes in defined spatiotemporal contexts and cell types; in this process, histone modifications act like a “dimmer switch,” enabling graded regulation through the addition or removal of marks by the corresponding enzymes. Histone lysine methylation modifies lysine residues via dedicated enzymes; by regulating methylation levels, histone lysine methyltransferases activate transcription, influence gene expression and neuronal structure and function, and can thereby lead to abnormalities in cognition.^9^ A large body of research supports a close association between histone lysine methylation and neurodevelopment as well as related disorders. Therefore, this review aims to summarize the roles of histone lysine methylation in neurodevelopment and disease, with the goal of providing new ideas and targets for the targeted treatment of NDDs.

## 2 HISTONE LYSINE METHYLATION

Histone modification is one of the most common categories within epigenetics.^10^ Histones (H), the principal protein components of nuclear chromatin, comprise five subtypes—H1, H2A, H2B, H3, and H4. Except for H1, the other four histones associate as dimers to form the nucleosome core.^11^ Histone modifications typically occur on the N‐terminal regions of histones and include methylation, acetylation, phosphorylation, lactylation, and others; these marks influence gene expression as well as neuronal structure and function.^12^ Histone methylation is a modification on lysine (K) or arginine (R) residues mediated by histone methyltransferases; lysine can be mono‐, di‐, or tri‐methylated (me1, me2, me3).^13^ Sites of histone lysine methylation include H3K4, H3K9, H3K27, H3K36, H3K79, and H4K20. By modifying specific amino‐acid residues, histone methylation determines whether transcription is activated or repressed.^14^ The activation or repression of gene transcription is dictated by the methylation site, degree, and pattern, as well as the genomic context in which the modification occurs.^15^

## 3 WRITERS AND ERASERS: KEY ENZYMES AND THEIR BIOLOGY

### 3.1 Histone H3K4 methyltransferases

Histone lysine methylation enzymes, or histone lysine methyltransferases (HMTs), are chiefly responsible for adding methyl groups to histones, thereby altering histone methylation states and influencing neuronal structure–function and gene transcription.^16^ Different HMTs methylate specific residues on specific histones. Histone H3K4 methyltransferases mainly include three types: KMT2/COMPASS‐like complexes,^17^ SET/COMPASS‐like complexes,^18^ and the DOT1 family.^19^ KMT2/COMPASS‐like complexes are a group of methyltransferases associated with hematologic malignancies; because members of KMT2 are observed in most blood cancers such as acute lymphoblastic leukemia (ALL) and acute myeloid leukemia (AML), they are also referred to as the myeloid/lymphoid or mixed‐lineage leukemia family^17^; Members include MLL1 (KMT2A), MLL2 (KMT2B), MLL3 (KMT2C), MLL4 (KMT2D), and MLL5 (KMT2E). Beyond histones, KMT2/COMPASS‐like complexes can also methylate non‐histone substrates in a methylation‐dependent manner.^20^ These complexes catalyze H3K4 methylation, a mark linked to gene activation.^21^ MLL1/MLL2 primarily catalyze H3K4me3 at promoters, whereas MLL3/MLL4 catalyze H3K4me1 at enhancers; MLL5 participates in the H3K4 regulatory network/complex functionality. Distinct subunits and cofactors (WD repeat‐containing protein 5 [WDR5], retinoblastoma binding protein 5 [RbBP5], absent‐small‐homeotic‐2‐like protein [ASH2L], dumpy‐30 [DPY‐30], etc.) determine site specificity and spatiotemporal selection across tissues. HMTs contain a conserved Su(var)3‐9, Enhancer‐of‐zeste, and Trithorax (SET) domain of ~130–150 amino acids that confers methyltransferase activity, and HMTs bearing a SET domain are termed SET‐domain group (SDG) proteins.^18^ Members of the SET/COMPASS‐like complexes include SETD1A (KMT2F), SETD1B (KMT2G), SETD2 (KMT3A), and SETD3–10. By shaping gene‐expression patterns and chromatin states, these complexes likely regulate chromatin architecture, gene expression, and processes such as learning and memory, thereby affecting neuronal development and function. SETD1A and SETD1B catalyze H3K4me3, while SETD2 and SETD3–10 catalyze H3K36me3 with specificity; in a transcription‐dependent manner, they enrich H3K36me3 within the coding regions of immediate early genes c‐Fos and c‐Jun.^22^, ^23^ SETD2 catalyzes H3K36me3,^24^ while SETD3 catalyzes all three methylation states of H3K4 and H3K36.^25^ Meanwhile, SETD3 is highly expressed in muscle tissue, and its overexpression can activate the transcription of muscle‐related genes such as myogenin, muscle creatine kinase, and myogenic regulatory factor 6 (MYF6), thereby inducing myogenic differentiation.^26^ SETD7 catalyzes H3K4me1, and SETD8 catalyzes H4K20me1.^27^ While this review focuses on H3K4, we retain interfaces to other methyl marks to explain cross‐modification coupling. The DOT1 family lacks a SET domain and consists of yeast DOT1 and its homolog DOT1L.^28^ DOT1L plays important roles in gene‐expression regulation and cell‐fate determination.^19^, ^28^ The DOT1 family catalyzes H3K79 methylation; quantitative Chromatin immunoprecipitation (ChIP) studies in the human genome show that H3K79me2 and H3K79me3 are associated with active gene transcription.^29^ Although most studied in hematopoiesis, DOT1‐mediated regulation has potential impacts on neurodevelopment and cell fate.^30^

### 3.2 Histone H3K4 demethylases

**Figure 1:**
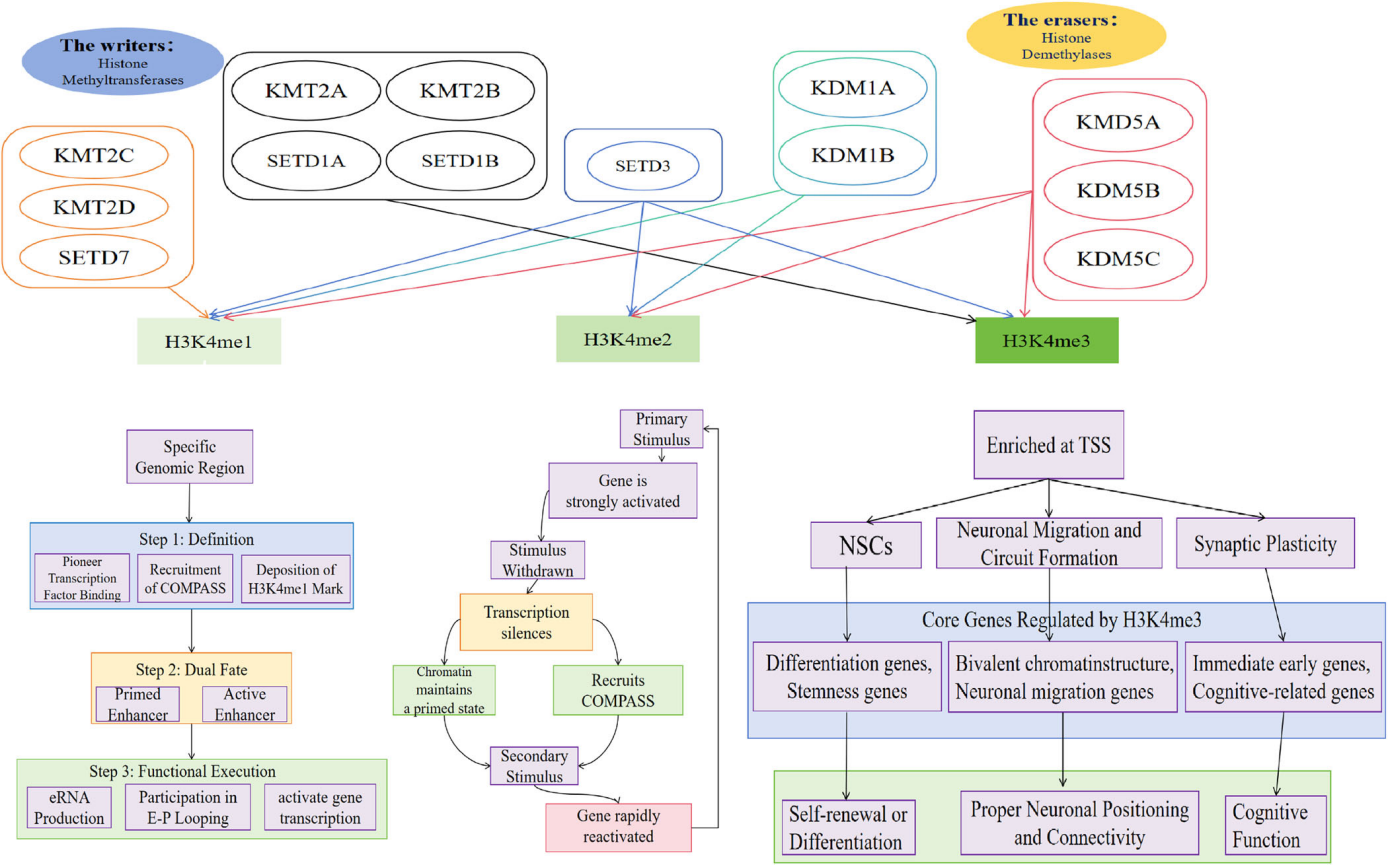
The dynamic regulatory network of H3K4 methylation. This illustration depicts how “writers” (KMT2/COMPASS‐like complexes, SET/COMPASS‐like complexes) and “erasers” (KDM1/5 families) coordinately regulate the three methylation states of histone H3K4:H3K4me1, H3K4me2, and H3K4me3. H3K4me1 serves as a mark for potential enhancers, though it requires cooperation with other factors to exert long‐range regulatory functions. H3K4me2 acts as a transcriptional “memory bookmark,” which explains how cells achieve faster responses to repeated signals and forms the basis for cellular memory and learning. H3K4me3 is enriched in promoter regions, where it regulates the expression of critical genes, thereby influencing key biological processes such as cell fate determination and neural function.

Histone H3K4 demethylases remove methyl marks from the H3K4 residue and are essential for maintaining normal gene expression. Because H3K4 methylation is a classic “gene‐activation” mark, demethylases counteract activation by removing these marks, rendering chromatin relatively closed and repressing transcription. The major known H3K4 demethylases fall into two families: the lysine‐specific demethylase (LSD) family and the Jumonji‐C (JmjC) family.^31^, ^32^ The LSD family uses flavin adenine dinucleotide (FAD) as a cofactor and removes methyl groups via an oxidation reaction. Because this chemistry removes one methyl group together with a hydrogen atom, LSD enzymes can demethylate only H3K4me2 and H3K4me1, but not H3K4me3.^33^ Members include lysine demethylase 1A (KDM1A) and lysine demethylase 1B (KDM1B). KDM1A, the first histone demethylase discovered (initially named LSD1), has broad functions in embryonic development, cell proliferation, and differentiation. It interacts with multiple complexes (e.g., CoREST, NuRD) to target specific genomic loci; in cancer, KDM1A can repress tumor‐suppressor genes.^34^, ^35^, ^36^ KDM1B is homologous to KDM1A; it can induce pluripotency gene expression, promote proliferation, enhance cellular reprogramming, and inhibit apoptosis.^37^ The JmjC family—also referred to as the lysine demethylase 5 (KDM5) family—requires Fe^2+^ and α‐ketoglutarate as cofactors and catalyzes demethylation of all three methylation states (H3K4me3/me2/me1) through a hydroxylation reaction.^32^ The KDM5 family comprises KDM5A, KDM5B, KDM5C, and KDM5D. Except for KDM5D, the other three members are enriched at promoters of active genes, where they fine‐tune expression amplitude and suppress “spurious activation.”^38^, ^39^, ^40^, ^41^ KDM5A regulates diverse processes—including learning and memory, neurogenesis, and dendritic morphology—by controlling H3K4 methylation.^42^ Its loss causes severe social and cognitive deficits, widespread transcriptomic dysregulation, and altered dendritic architecture.^41^ Mirroring human life‐stage patterns, KDM5B expression is markedly higher in the embryonic brain than in the adult brain; consequently, embryonic KDM5B‐mutant mice show extensive differential gene expression, with functions enriched in learning and memory, brain development, and synaptic structure.^40^, ^43^ KDM5C and KMT2A are broadly expressed across brain regions, suggesting that they may jointly maintain H3K4me3 homeostasis and cognitive function.^39^ Experimental evidence shows that KDM5C knockout mice display cognitive deficits and synaptic abnormalities, similar to those observed in KMT2A‐mutant mice. Notably, double mutation of KMT2A and KDM5C markedly reverses dendritic defects and learning impairments, and partially restores the altered transcriptome and H3K4me3 landscape.^44^ Because loss of KMT2A reduces H3K4me3 while loss of KDM5C causes excessive H3K4me3 accumulation, a balance between these enzymes may represent a shared epigenetic mechanism that preserves learning and memory capacities and synaptic structure (Figure 1).

## 4 HISTONE LYSINE METHYLATION AND NEURODEVELOPMENT

### 4.1 Effects of histone lysine methylation on neurodevelopment

Histone lysine methylation is maintained by a dynamic balance between histone lysine methyltransferases and demethylases. Mono‐, di‐, and tri‐methylation of H3K4 are generally associated with promoter activation. H3K4me1 often marks enhancers. The H3K4me1 “writer” KMT2D can specifically open the WNT3A enhancer in roof plate–like cells, thereby sustaining a WNT microenvironment essential for the normal development of neural crest and neural progenitor cells.^45^ In mouse embryonic stem cell neural differentiation, H3K4me1 also facilitates enhancer–promoter (E–P) interactions to drive neural lineage commitment.^46^ H3K4me2 has been studied less extensively but plays an important role in transcriptional memory—a phenomenon in which prior exposure to a stressor primes a stronger response to subsequent similar stress, enabling better adaptation to environmental changes.^47^, ^48^ Under the initial stimulus, the promoter regions of specific genes are rapidly activated. The COMPASS complex is recruited to these regions and catalyzes the deposition of H3K4me3, thereby establishing a chromatin environment permissive for active transcription and markedly increasing gene expression. After the stimulus is withdrawn, transcription gradually becomes silenced, and H3K4me3 levels decline; however, the promoter retains a residual level of H3K4me2, which is deposited on chromatin as an “epigenetic bookmark.” The presence of H3K4me2 indicates that the gene was previously active and helps maintain chromatin in a “poised” open conformation, allowing transcription‐associated factors to remain bound or retained at low levels. Consequently, upon re‐exposure to the same or a similar stimulus, promoters carrying the H3K4me2 bookmark can be recognized and respond more rapidly: the COMPASS complex is recruited more quickly to H3K4me2‐marked regions, and H3K4me2 also serves as a platform for the binding of other chromatin‐remodeling complexes or transcription factors, thereby accelerating the re‐establishment of H3K4me3. Ultimately, this leads to faster and stronger transcriptional reactivation of the gene.^49^, ^50^, ^51^ H3K4me3 is enriched at transcription start sites, where it associates with the transcription preinitiation complex and RNA polymerase II to promote transcription initiation and elongation. H3K4me3 can also recruit co‐activators, loosening chromatin, increasing DNA accessibility, and boosting the expression of learning‐ and memory‐related genes such as activity‐regulated cytoskeleton‐associated protein (Arc), Fos, and neuronal PAS domain protein 4 (Npas4). Moreover, H3K4me3 is enriched at brain‐derived neurotrophic factor (BDNF) promoters, enhancing hippocampal synaptic plasticity and thereby facilitating learning and memory.^52^, ^53^, ^54^, ^55^ Collectively, these studies indicate that histone lysine methylation plays a crucial role in neurodevelopment.

### 4.2 Crosstalk between histone lysine methylation and other epigenetic mechanisms during neurodevelopment

**Figure 2:**
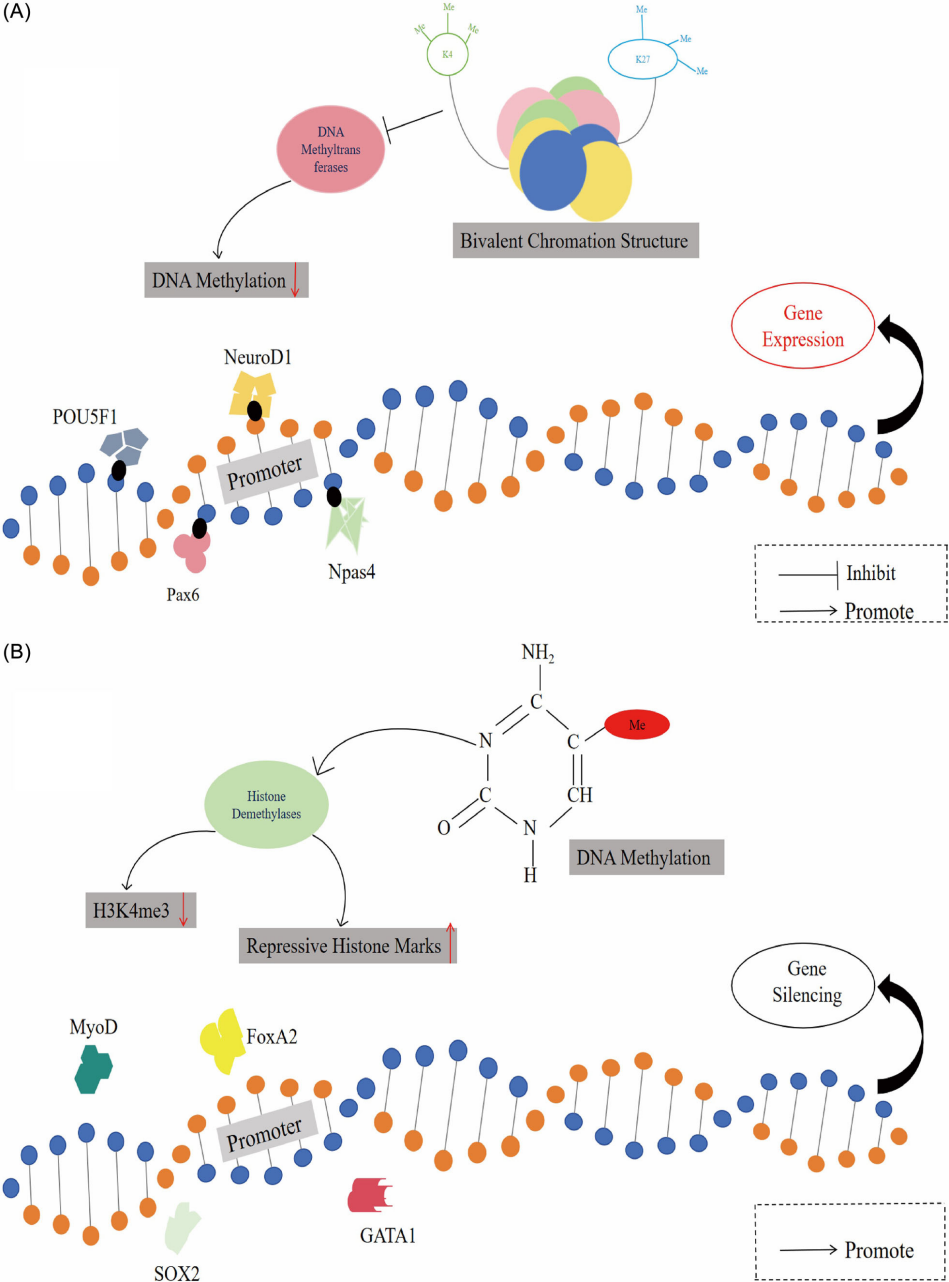
The “antagonistic” dialog between H3K4me3 and DNA methylation. (A) The bivalent chromatin structure in the promoter region maintains a low DNA methylation environment, allowing transcription factors to bind to the promoter and promote gene expression. (B) Conversely, a high DNA methylation environment recruits histone demethylases, transcription factors fail to bind to the promoter, and gene silencing occurs.

Histone lysine methylation does not act in isolation; together with DNA methylation and histone acetylation, it forms a precise, interactive network that co‐regulates the expression of neurodevelopmental genes. DNA methylation—the first epigenetic modification to be discovered—refers to the selective addition of a methyl group to specific DNA bases by DNA methyltransferases, most commonly to cytosines within CpG islands.^5^ Extensive crosstalk exists between DNA methylation and histone methylation. In most cases, DNA methylation and H3K4me3 cannot coexist within the same genomic region, especially at gene promoters. As an activating mark, H3K4me3 is enriched at promoters of active genes, indicating a “poised for transcription” or “actively transcribing” state and promoting gene expression.^56^ By contrast, dense DNA methylation at promoters blocks transcription factor binding and recruits repressive proteins, thereby “turning off” the gene and culminating in gene silencing.^57^ Neurodevelopment is a highly ordered process in which distinct epigenetic modifications assume different roles. In neural stem cells (NSCs), promoters of pluripotency‐related transcription factors (particularly POU class 5 homeobox 1 [POU5F1/OCT4] and paired box 6 [PAX6]) display a bivalent chromatin configuration enriched for both H3K4me3 and H3K27me3. Within this bivalent state, H3K4me3 serves to prevent DNA‐methylation‐mediated silencing of these pluripotency factors, providing flexibility for their rapid activation after differentiation.^58^ Once NSCs begin to differentiate into specific neuronal or glial lineages, the epigenetic landscape undergoes dramatic remodeling. For genes that need to be activated (e.g., the neuron‐specific transcription factor NeuroD1^59^), the presence of H3K4me3 can suppress the activity of DNA methyltransferases at these regions, thereby ensuring that the promoter remains in a low–DNA methylation state and enabling sustained active gene expression.^60^ H3K4me3 inhibits DNA methyltransferases mainly by preventing the ATRX‐DNMT3‐DNMT3L (ADD) domain of DNMT3A/3L from binding to chromatin, or indirectly by allowing CXXC finger protein 1 (CFP1) to help maintain an unmethylated CpG state within H3K4me3‐enriched regions, thereby suppressing DNA methylation. De novo DNA methylation is mediated by DNMT3A together with its cofactor DNMT3L. This complex contains an ADD domain that interacts with unmethylated H3K4 (H3K4me0). Through intramolecular interactions, the ADD domain can block the methyltransferase catalytic domain; binding to H3K4me0 relieves this autoinhibition.^61^, ^62^, ^63^, ^64^ In simple terms, DNA methylation by the DNMT3A/3L complex depends on cooperation between the ADD domain and H3K4me0. Therefore, when the complex encounters regions enriched in the active mark H3K4me3, H3K4me3 directly inhibits the ADD domain, preventing it from functioning properly. As a result, the DNMT3A/3L complex fails to catalyze DNA methylation at these loci, and DNA methylation cannot be established there. The CXXC domain is a zinc‐finger motif found in many proteins. Its key feature is the specific recognition and binding of unmethylated CpG dinucleotides, with extremely low—or even no—affinity for methylated CpGs. CFP1 is a CXXC domain, containing protein and a core component of the SET/COMPASS and MLL/COMPASS complexes. CFP1 can promote H3K4me3 deposition at these regions and establishes a reinforcing loop—namely, CFP1 recruitment via the CXXC domain to unmethylated CpG sites promotes H3K4me3 establishment, which in turn antagonizes DNA methylation—thereby indirectly suppressing DNA methylation at target regions.^65^, ^66^ In contrast, for genes that need to be permanently silenced (e.g., pluripotency transcription factors and lineage‐specifying genes), DNA methylation can recruit histone demethylases to remove H3K4 methylation and to add repressive histone marks, resulting in a stable, heritable silent state.^67^ This silencing can be maintained through cell division and transmitted to daughter cells, thereby ensuring the stability of cell fate (Figure 2).

**Figure 3:**
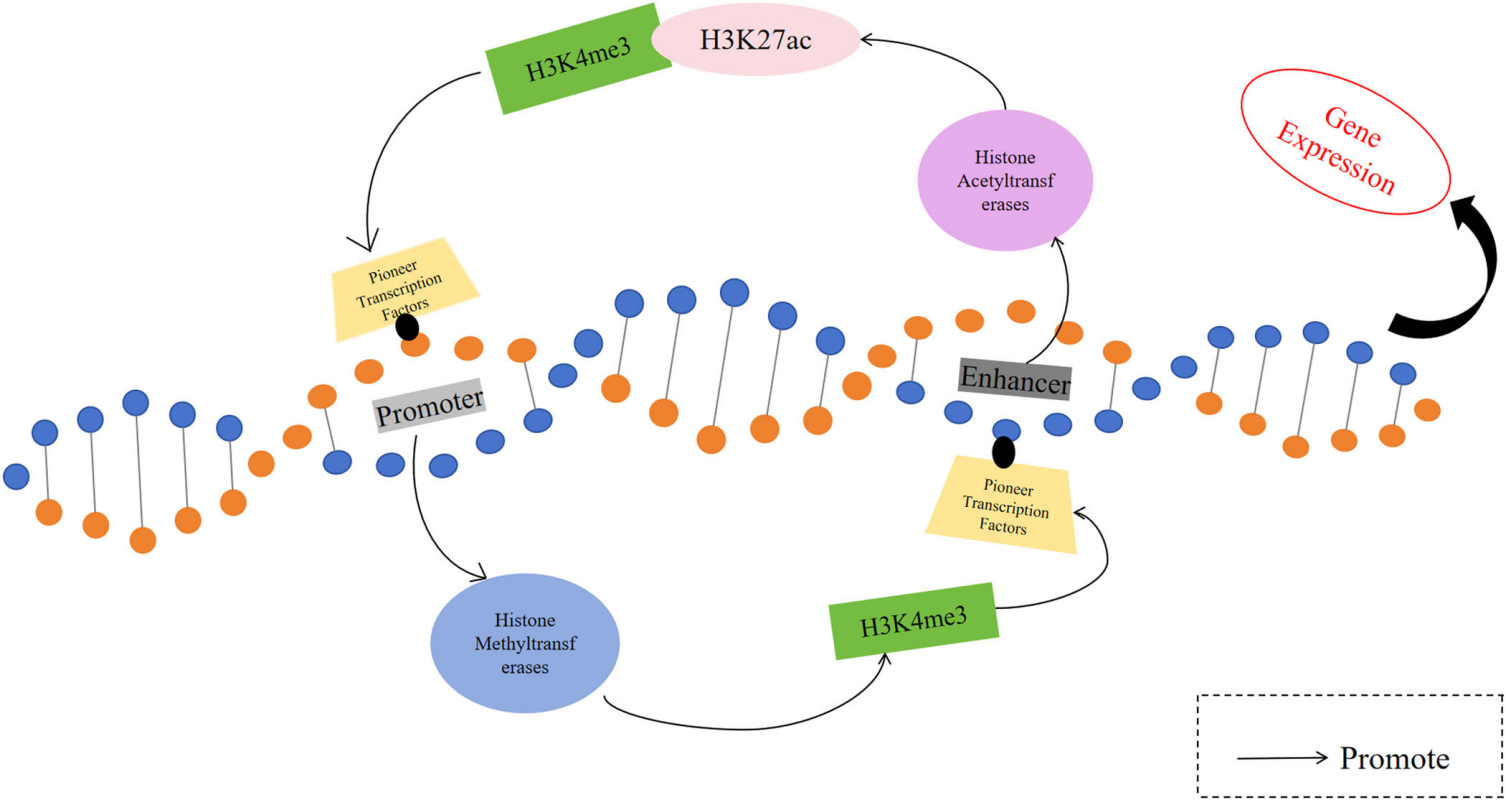
The “synergistic” interaction between H3K4me3 and histone acetylation. H3K4me3 and histone acetylation H3K27ac form a “positive feedback loop” to promote gene expression.

Histone acetylation and H3K4me3 act in concert during neurodevelopment, forming a powerful gene‐activation duo while operating at different layers. Histone acetylation refers to the addition of an acetyl group to lysine residues on histone tails catalyzed by histone acetyltransferases.^68^ By neutralizing the positive charge of histones, acetylation loosens chromatin structure, opening access for transcription factors and complexes.^69^ H3K4me3 and histone acetylation are strongly positively correlated and frequently reinforce each other's establishment and maintenance. H3K4me3 provides specificity and stability for transcription by precisely marking promoters of genes destined for activation, whereas histone acetylation provides accessibility and efficiency by creating an open chromatin environment that allows H3K4me3‐primed transcription factors to bind DNA and initiate transcription smoothly.^70^, ^71^, ^72^ During neurodevelopment, the two play distinct roles. In NSCs, the enhancer and promoter regions of key fate‐determining genes are often simultaneously enriched for H3K4me3 and H3K27ac. This “H3K4me3 + H3K27ac” combination keeps these critical genes in an “initiated” or “poised” state: chromatin remains open, and the genes are expressed at low basal levels or can be readily induced. This configuration endows NSCs with the capacity to respond rapidly to differentiation cues.^73^, ^74^ Subsequently, upon receiving differentiation signals, cells must rapidly and extensively reprogram their gene expression landscape; therefore, promoters and enhancers of genes that need to be activated quickly acquire high levels of H3K4me3 and histone acetylation. Pioneer transcription factors bind to these regions to initially open local chromatin architecture and recruit histone acetyltransferases, thereby increasing acetylation levels. The resulting open chromatin environment further facilitates the binding and activity of H3K4 methyltransferases, elevating H3K4me3 levels. In turn, the establishment of H3K4me3 feeds back to recruit additional acetyltransferases, forming a positive‐feedback loop that progressively reinforces chromatin accessibility and transcriptional activity, ensuring stable and high‐level expression of pioneer transcription factors.^75^, ^76^, ^77^ Pioneer transcription factors can bind partially compacted chromatin and locally unwind nucleosomes via their DNA‐binding domains, or recruit chromatin‐remodeling complexes such as SWI/SNF, thereby creating an initial accessibility window for histone acetyltransferases.^75^ Pioneer transcription factors include Achaete‐Scute family bHLH transcription factor 1 (ASCL1), neurogenin3 (NGN3), NeuroD1, SRY‐box transcription factor 2 (SOX2), and PAX6. ASCL1 belongs to the basic helix–loop–helix (bHLH) transcription factor family, binds specific DNA sequences (e.g., the E‐box motif 5′‐CANNTG‐3′), and achieves efficient DNA binding through dimerization with other bHLH proteins. As a pioneer factor, ASCL1 is uniquely capable of accessing “closed” chromatin, opening chromatin structure to allow other transcription factors to bind and activate neuronal gene expression, thereby initiating neurodevelopmental or regenerative programs.^78^, ^79^ NGN3 is also a bHLH transcription factor that can recognize specific DNA sequences within compact chromatin and initiate chromatin remodeling, rendering the locus transcriptionally competent and laying the foundation for subsequent NeuroD1 binding and gene activation.^80^ NeuroD1 plays important roles in nervous system development and disease therapy. NeuroD1, likewise a bHLH factor, forms heterodimers with E proteins and binds E‐box elements (e.g., CANNTG) in target genes to regulate transcription. It exerts dual functions in neurodevelopment: on the one hand, it is a key driver of neuronal differentiation from neural progenitors; on the other hand, it can induce direct reprogramming of non‐neuronal cells (e.g., astrocytes) into functional neurons.^81^, ^82^ SOX2 cooperates with factors such as OCT4 and NANOG to bind enhancers of numerous pluripotency genes (including its own), maintaining an open chromatin state enriched in H3K4me3 and H3K27ac and preventing the encroachment of repressive modifications, thereby safeguarding stemness and self‐renewal capacity.^83^ During neural differentiation, SOX2 expression is precisely regulated; in specific spatiotemporal contexts, it partners with different factors to actively remodel chromatin and initiate neural‐specific gene programs.^84^ In NSCs, PAX6 and SOX2 constitute a classic cooperative pioneer pair that co‐occupies many target genes, maintaining cells in a state that is “plastic yet primed for a neural fate.”^85^ Accordingly, within the positive‐feedback loop between H3K4me3 and H3K27ac, the sequential recruitment of specific pioneer transcription factors and histone acetyltransferase subtypes can be described as follows: first, key genes are bound by factors such as SOX2/PAX6 to maintain “poised” enhancers, where moderate levels of H3K27ac and H3K4me1 are already present. As differentiation signaling persists and induces the expression of pioneer factors such as ASCL1/NeuroD1, these factors bind target sites and recruit the histone acetyltransferases p300/CBP, rapidly increasing H3K27ac levels and enabling initial chromatin opening. The acetylated chromatin then recruits the WDR5/MLL complex to catalyze the establishment of H3K4me3. Thereafter, H3K4me3 and H3K27ac mutually reinforce each other through a positive‐feedback loop, while recruiting a more comprehensive transcriptional machinery to drive the gene into a stable high‐expression state.^86^, ^87^, ^88^, ^89^ In contrast, at genes that need to be silenced, histone deacetylases and histone demethylases are recruited to erase both H3K27ac and H3K4me3 and may introduce repressive modifications, leading to chromatin compaction and stable transcriptional silencing^90^ (Figure 3).

In summary, histone lysine methylation—especially H3K4me3 as a pivotal activating mark—does not act alone in regulating gene expression during neurodevelopment. Instead, it operates through a dynamic, synergistic, and mutually counterbalancing epigenetic network with DNA methylation and histone acetylation. The core logic of this network is reflected on two levels: an “antagonistic” dialog with DNA methylation and a “synergistic” interplay with histone acetylation. In the antagonistic dialog, H3K4me3 and DNA methylation are mutually exclusive at gene promoters, safeguarding the promoters of pluripotency and neuron‐specific genes from silencing while ensuring long‐term stability of cell fate. In the synergistic interplay, H3K4me3 and histone acetylation (e.g., H3K27ac) form a potent positive feedback loop. Their co‐occurrence at enhancers and promoters of key neurodevelopmental genes—particularly during differentiation—leads to reciprocal reinforcement that elevates and stabilizes gene expression in an open chromatin state, thereby precisely driving the cascades of neurodevelopment. Consequently, the proper execution of neurodevelopment depends on the fine‐tuned regulation of this epigenetic network comprising histone methylation, DNA methylation, and histone acetylation. Dysregulation of any component can upset the balance of the entire network, disrupt transcriptional programs, and likely underlie the pathogenesis of numerous NDDs.

### 4.3 Epigenetic programming during neurodevelopment

#### 4.3.1 NCSs and bivalent chromatin

Histone lysine methylation not only influences transcriptional activity but, through precise spatiotemporal control, participates in multiple key stages of neurodevelopment—most notably NSCs fate decisions, neuronal migration and circuit formation, and synaptic plasticity and learning–memory. A foundational aspect is the balance between self‐renewal and differentiation of NSCs, which underpins normal brain development.^91^ Bivalent chromatin—marked by H3K4me3 and H3K27me3—plays a pivotal role in maintaining genes in a reversible “on/off” state and dynamically modulates NSCs' proliferation and differentiation.^92^, ^93^, ^94^ In NSCs, promoters of many developmentally critical genes are concurrently enriched for H3K4me3 (activating) and H3K27me3 (repressive) marks.^95^ H3K4me3 is enriched in the subgranular zone of primary neurogenic niches, whereas H3K27me3 is found mainly in the granule cell layer. Elevated H3K4me3 promotes proliferation and development of neonatal mouse NSCs and biases differentiation toward GABAergic neurons; in sharp contrast, elevated H3K27me3 drives NSCs into a quiescence‐like state.^93^ The coexistence of these seemingly opposing states endows NSCs with maximal plasticity and flexibility, enabling responses to diverse differentiation cues and trajectories. As NSCs differentiate into specific neuronal lineages, this bivalent configuration is resolved: selective removal of methyl marks determines whether a gene is ultimately activated or permanently silenced. For genes that must be activated (e.g., lineage‐specific genes), the histone demethylase KDM6B is recruited to target loci, erasing H3K27me3, thereby consolidating H3K4me3 and likely recruiting additional co‐activator complexes. The promoter thus transitions from a “bivalent” to a purely “active” state, with robust gene activation that drives commitment to defined neuronal lineages.^96^, ^97^ Conversely, for genes requiring permanent silencing (such as POU5F1, SOX2, and Nanog), members of the KDM5 family are recruited to remove H3K4me3. Loss of this activating mark allows further consolidation of H3K27me3 and likely recruitment of DNA methyltransferases, establishing a more stable repressive state. The promoter shifts from “bivalent” to purely “repressed” the gene is permanently shut down, and cell fate proceeds irreversibly along its prescribed course.^98^, ^99^

#### 4.3.2 Neuronal migration and circuit formation

The migration of neurons from their birthplace to specific destinations is a prerequisite for building complex neural networks and for regulating circuit formation and function across the lifespan; this process proceeds through coordinated morphological and gene‐expression changes.^100^ The bivalent chromatin configuration composed of H3K4me3 and H3K27me3 also functions during neuronal migration. This bivalency is commonly found on neuronal genes in granule cell progenitors (GCPs) and undergoes gene expression‐linked dynamics during migration and circuit assembly. Neuronal development is governed by gene‐expression programs that time the progression of neuronal progenitors as they undergo glia‐guided migration and circuit formation.^101^ Cerebellar granule cells (GCs) provide an ideal model for studying these processes because they represent a large, relatively homogeneous progenitor population that passes through stereotyped, well‐defined developmental stages before becoming fully functional central nervous system (CNS) neurons.^95^, ^102^ After sustained proliferation in the external granular layer, GCPs exit the cell cycle and enter differentiation; during this phase, they migrate along radial glial fibers, gradually acquire a bipolar morphology, extend parallel fibers, and commence glia‐guided migration.^103^ Inhibiting the H3K27 methyltransferases EZH1 and EZH2 in vitro and in vivo markedly alters the expression of bivalent genes, downregulates migration‐related genes, suppresses glia‐guided migration, and accelerates terminal differentiation.^101^ Thus, histone bivalency is essential for regulating neuronal migration and circuit formation.

#### 4.3.3 Synaptic plasticity and learning–memory

As noted earlier, H3K4me3 plays a prominent role in synaptic plasticity and learning–memory. Learning and memory engage extensive, interconnected neural circuits in which neurons transmit signals to one another via synapses. Activity‐dependent changes in synaptic morphology or transmission efficacy—manifesting as long‐term potentiation (LTP) or long‐term depression—constitute the cellular foundation of learning and memory.^104^ H3K4me3 influences plasticity by regulating the expression of plasticity‐related genes such as BDNF and Arc. Enrichment of H3K4me3 at BDNF promoters increases dendritic spine density in hippocampal neurons and enhances learning and memory.^55^, ^105^ Arc is rapidly induced by neuronal activity and modulates synaptic plasticity by controlling downstream secondary‐response genes.^106^ Enzymes that regulate H3K4 also shape synaptic plasticity. Forebrain‐specific deletion of the H3K4 methyltransferase MLL1 reduces H3K4me3 in striatal/nucleus accumbens neurons and weakens LTP.^107^ KDM5B functions as a key regulator of hippocampal synaptic plasticity; knockout mice exhibit deficits in hippocampus‐dependent learning/memory and in LTP.^40^ Demethylases LSD1 and KDM5C dynamically tune H3K4me1/2/3 levels to modulate activity‐dependent gene expression, thereby fine‐adjusting synaptic structure and function.^108^ Through such epigenetic mechanisms, external experience is converted into lasting changes in neural connectivity—the basis of higher cognitive functions.

## 5 DYSREGULATION OF METHYLATION AND NDDS

When the molecular and cellular mechanisms described above go awry, they directly lead to clinically evident NDDs. Below, we discuss representative diseases closely linked to abnormal histone lysine methylation.

### 5.1 Kabuki syndrome

Kabuki syndrome is a congenital genetic disorder caused by mutations in KMT2D, most commonly affecting infants and children.^109^, ^110^ KMT2D regulates cell development, differentiation, and normal physiological functions, and plays an essential role in cell‐type‐specific gene expression during neuronal growth and differentiation.^111^, ^112^ Mutations in KMT2D disrupt H3K4me1 and impair normal growth and development. Animal studies support this: deletion of KMT2D leads to embryonic lethality in mice around embryonic day 9.5.^113^ In addition, KMT2D mutations cause defects in neural crest differentiation and premature neuronal differentiation skewed toward inhibitory neuronal lineages, which may partly explain neural‐crest‐related manifestations such as intellectual disability in patients with Kabuki syndrome.^45^ Experimental data further show that the KMT2D^+/βGeo^ mouse model exhibits impaired adult neurogenesis in the dentate gyrus and concomitant hippocampal memory abnormalities due to disrupted hippocampal neurogenesis.^114^, ^115^ There are pharmacological approaches that can ameliorate cognitive deficits caused by KMT2D mutations. In KMT2D^+/βGeo^ mice, adult neurogenesis deficits and hippocampal memory impairments are improved by AR‐42, a histone deacetylase inhibitor that restores H3K4me3 levels and rescues dentate gyrus structure and function.^116^ Oral administration of TAK‐418 (a KDM1A inhibitor) increases adult neurogenesis and the number of doublecortin (DCX^+^) cells in the dentate granule cell layer; it also elevates global H3K4 mono‐, di‐, and tri‐methylation in KMT2D^+/βGeo^ mice, thereby improving visuospatial learning and memory deficits.^114^

### 5.2 Wiedemann–Steiner syndrome

Wiedemann–Steiner syndrome (WSS) is a rare hereditary disorder caused by mutations in KMT2A.^17^, ^117^ As an H3K4me3‐specific methyltransferase, KMT2A is critical for memory: its loss leads to severe defects in memory consolidation and formation in contextual fear conditioning.^118^ Compared with wild‐type mice, KMT2A mutant mice show more pronounced memory impairments in the Morris water maze; in cortical neurons, multiple neuropsychiatric susceptibility genes are among the most affected; and altered dendritic morphology in the prefrontal cortex leads to synaptic plasticity defects.^39^, ^44^ These findings underscore the importance of KMT2A in maintaining neuronal structure–function and cognitive abilities. Although no definitive therapy exists for WSS, KDM5C emerges as a potential target: during neuronal maturation, KDM5C regulates expression of synaptic plasticity genes and enhances activity‐dependent enhancers by modulating immediate‐early gene expression. Neurons lacking KDM5C cannot properly shape their epigenetic landscape or eliminate spurious transcription, which may contribute to neuropsychiatric symptoms^119^; thus, targeting KDM5C could open new therapeutic avenues for WSS.

### 5.3 Autism spectrum disorders

Autism spectrum disorder (ASD) is a highly heritable neurodevelopmental condition with unclear etiology. Whole‐exome sequencing has identified two ASD‐associated genes: KMT2E and regulating synaptic membrane exocytosis 1 (RIMS1).^120^ KMT2E belongs to a family of enzymes critical for transcriptional control via histone methylation; its variants are associated with NDDs typically characterized by intellectual disability, hypotonia, gastrointestinal issues, and mild facial dysmorphisms.^121^ Li et al. reported that KMT2E haploinsufficient mice exhibit social deficits accompanied by anxiety; positron emission tomography revealed markedly reduced glucose metabolism in the amygdala and evidence of disordered neuronal development.^120^ As there are no medications that treat the core deficits of ASD, deeper investigation of how KMT2E loss affects behavior and the nervous system may inform new therapeutic strategies.

### 5.4 Schizophrenia

Whole‐exome sequencing of over 200 patients with schizophrenia identified mutations in SETD1A,^122^ implicating SETD1A in disease etiology. Psychiatric disorders often involve disruptions of neuronal networks that may originate early and persist throughout life. SETD1A mediates H3K4me3 and plays a key role in neuronal development and maturation, particularly in the timing and amplitude of gene expression.^123^ Loss of SETD1A function likely causes aberrant gene expression, preventing appropriate gene activation during brain development; such dysregulation can miswire neuronal networks and ultimately produce the brain dysfunction characteristic of schizophrenia.^69^, ^124^ In animal studies, SETD1A^+/−^ mice display working‐memory impairments and cortical synaptic plasticity deficits, along with altered axonal branching and cortical synapse dynamics.^22^, ^125^ SETD1A binds both promoters and enhancers, and myocyte enhancer factor 2 (Mef2) functionally overlaps with SETD1A; co‐expression may produce synergistic effects on gene regulation that contribute to cognitive impairments.^126^ Although no targeted therapies have yet emerged for schizophrenia, Mukai et al.^22^ showed that restoring SETD1A expression in adult mice can rescue cognition and network defects arising from SETD1A mutations—suggesting potential reversibility and the promise of adult‐stage interventions.

### 5.5 Shared and distinct disease mechanisms: Convergence and divergence of dysregulated H3K4 methylation in NDDs

The four NDDs caused by loss‐of‐function of distinct H3K4 methyltransferases—KMT2A, KMT2D, KMT2E, and SETD1A—exhibit heterogeneous clinical manifestations. However, their molecular and cellular pathologies reveal profound commonalities, while also displaying unique disease trajectories driven by spatiotemporal expression specificity and differences in genomic targeting.

#### 5.5.1 Shared mechanisms: Epigenetic dysregulation of synaptic plasticity and neurogenesis gene networks

The causative genes in these four disorders all encode core catalytic subunits of COMPASS‐like complexes responsible for depositing H3K4me3 at specific genomic regions. Their mutations converge on a shared downstream consequence: failure of epigenetic programming of gene networks governing synaptic plasticity and neurogenesis. Although each enzyme has its own preferences, genome‐wide analyses indicate that they collectively regulate a core set of genes tightly linked to neurodevelopment. Multiple studies suggest that the establishment and maintenance of H3K4me3 at promoters or enhancers of postsynaptic scaffold genes (e.g., SHANK3),^127^ ion channel subunit genes (e.g., GABA receptor subunits),^93^ and activity‐dependent immediate early genes (e.g., Arc and Fos)^52^, ^55^ depend heavily on these methyltransferases. In both KMT2A mutant mice and SETD1A haploinsufficient mouse models, reduced H3K4me3 levels and transcriptional downregulation of such genes have been observed in cortical neurons, directly correlating with synaptic plasticity defects, such as impaired LTP and neuronal morphological abnormalities, including reduced dendritic complexity.^39^, ^44^, ^123^, ^125^ This shared targeting helps explain why different syndromes commonly co‐present with intellectual disability, learning and memory deficits, and abnormal social behaviors. Moreover, H3K4me3 is a key epigenetic mark that determines cell fate decisions and the timing of differentiation. KMT2A, KMT2D, and SETD1A are all highly expressed in specific neural stem/progenitor populations, and their inactivation leads to a common defect: an inability to establish the correct H3K4me3 landscape in the right cell types at the right developmental time. This results in two major outcomes. First, differentiation is blocked or aberrant. In Kabuki syndrome, KMT2D mutations cause differentiation defects in neural crest cells, leading to craniofacial skeletal abnormalities and autonomic nervous system maldevelopment; in parallel, persistent impairment of adult neurogenesis in the hippocampal dentate gyrus compromises memory formation.^45^, ^114^ Second, neuronal maturation is disrupted. In SETD1A‐related schizophrenia models, cortical neurons can be generated, but programs governing axon guidance, dendritic morphogenesis, and maturation of functional synaptic connectivity are misregulated, ultimately leading to improper assembly of neural networks.^22^, ^125^ Together, these findings underscore that H3K4me3 regulation is equally critical for both the “quantity” of neurons produced and the “quality” of their maturation.

#### 5.5.2 Distinct mechanisms: Cell‐type vulnerability and developmental windows shape clinical heterogeneity

Despite these shared mechanisms, disease‐specific clinical features arise from the spatiotemporal expression patterns of each causative gene and its unique downstream target set. One major factor is cell‐type‐specific vulnerability. KMT2D plays an irreplaceable role in craniofacial neural crest cells; its mutation selectively disrupts enhancer H3K4me1 states at craniofacial development‐related genes (e.g., TFAP2B and genes in the EDN1 pathway), leading to the characteristic facial dysmorphism and congenital heart defects of Kabuki syndrome—features that clearly distinguish it from disorders primarily affecting the cerebral cortex.^45^, ^111^ KMT2A is highly expressed in hippocampal and prefrontal cortical neurons in mice, where it specifically regulates gene programs involved in memory consolidation and higher‐order cognition; its mutation induces dendritic abnormalities and synaptic plasticity deficits in these regions, closely matching the severe intellectual disability and memory impairment in WSS, while craniofacial anomalies are relatively mild.^118^ SETD1A is essential in developing cortical projection neurons and regulates genes required for cortical lamination and circuit formation; haploinsufficiency results in altered intrinsic excitability and abnormal network synchrony in cortical neurons, which more directly relates to the core symptoms of schizophrenia, including working memory deficits and thought disorganization.^22^, ^126^ Another key contributor is disease‐specific remodeling of the epigenetic landscape. KMT2D is primarily enriched at enhancers and catalyzes H3K4me1, whereas KMT2A and SETD1A predominantly catalyze H3K4me3 at promoters. Accordingly, KMT2D mutations primarily disrupt enhancer activity and cell‐type‐specific gene regulation, while KMT2A and SETD1A mutations more directly impair transcription initiation of critical protein‐coding genes. In addition, each methyltransferase engages distinct protein‐protein interaction networks. For example, SETD1A has been reported to cooperate with the transcription factor Mef2 to regulate synaptic genes,^126^ whereas KMT2D interacts closely with acetyltransferases such as p300 at enhancers.^88^ Mutations disrupt these specific cooperative relationships, resulting in divergent gene expression dysregulation profiles across disorders.

## 6 ANIMAL MODELS AND KEY TECHNOLOGICAL ADVANCES

A deep understanding of how histone lysine methylation contributes to NDDs relies heavily on innovative research models and cutting‐edge technologies. From in vivo animal models to ex vivo cellular systems and high‐throughput sequencing, these tools together have propelled rapid progress in the field.

### 6.1 In vivo models

The most common approach is gene knock‐out/knock‐up mouse models. Whole‐body knockouts via homologous recombination or gene activation via the CRISPR/dCas9‐SAM system provide the most direct means to interrogate gene function. For example, conditional knockout of KMT2D has revealed embryonic lethality early in development, whereas nervous‐system‐specific deletion faithfully recapitulates the neurogenesis defects and memory impairments observed in patients with Kabuki syndrome.^113^, ^114^, ^115^ Similarly, SETD1A heterozygous knockout mice exhibit working‐memory deficits and cortical synaptic abnormalities, providing key in vivo evidence for a causal link to schizophrenia.^22^ CRISPR activation plasmids employ the synergistic activation mediator (SAM) complex, assembled at a 1:1:1 ratio: a plasmid encoding a nuclease‐dead Cas9 (dCas9; D10A and N863A) fused to the VP64 transactivation domain; a plasmid encoding the MS2‐p65‐HSF1 fusion protein; and a plasmid encoding a 20‐nt gRNA targeting the promoter of the gene of interest. The plasmids are then packaged into adeno‐associated viral vectors.^128^, ^129^ The CRISPR/dCas9‐SAM technique is designed specifically for gene “knock‐up” (activation): it does not perform gene knockout or knock‐in/correction. Instead, dCas9 precisely localizes without cutting DNA and recruits the transcriptional machinery via potent activation domains to markedly boost endogenous gene expression—useful for restoring or enhancing genes with haploinsufficiency. Point‐mutation and humanized mouse models are also widely used. Compared with full knockouts, introducing patient‐specific point mutations (e.g. those that impair the catalytic activity of the SET domain) more precisely models human disease. Such models can disentangle enzymatic, catalytic‐activity‐dependent roles from non‐catalytic functions, and they better mirror clinical states at molecular, cellular, and phenotypic levels—crucial for developing drugs that target catalytic activity.^130^, ^131^ Non‐mammalian models are likewise valuable. Drosophila and zebrafish offer ease of genetic manipulation, relatively simple nervous systems, and short life cycles, making them ideal for large‐scale genetic screens and initial mechanistic exploration.^132^, ^133^

### 6.2 Brain organoids and cellular models

Human induced pluripotent stem cell technology enables reprogramming of patient somatic cells into pluripotent stem cells and subsequent differentiation into three‐dimensional brain organoids. Unlike traditional two‐dimensional culture, organoids self‐organize and differentiate within a 3D scaffold under defined conditions.^134^, ^135^ This captures aspects of early human brain development that animal models cannot fully recapitulate, allowing direct observation of early regionalization, neurogenesis, and neuronal migration. Crucially, patient‐derived organoids retain individual genetic backgrounds and can reproduce early developmental events, thereby bridging interspecies gaps. They serve as “miniaturized in vitro models” to study how patient‐specific mutations perturb human neurodevelopment. For example, using organoids derived from Kabuki syndrome patient iPSCs, SHAN and colleagues directly observed KMT2D‐loss‐induced neural crest differentiation defects and dysregulation of enhancer activity.^45^

### 6.3 Multi‐omics and chromatin atlases

Chromatin mapping is now a mainstay. ChIP‐seq and derivative techniques (e.g. CUT&Tag) are central for generating genome‐wide maps of histone modifications.^136^, ^137^ Although all cells in an individual share the same DNA, they differ because distinct gene programs define cell identity and tissue type. Chromatin atlas technologies reveal these programs and, importantly, identify regulatory sequences within the >90% noncoding portion of the human genome that control gene on/off states. By comparing the distribution of regulatory chromatin features between disease and control models, researchers can pinpoint dysregulated genes and enhancers, greatly aiding mechanistic understanding of pathogenesis.^138^ Integrated multi‐omics analyses are also frequently applied—combining genomics, epigenomics, transcriptomics, proteomics, and metabolomics to reconstruct comprehensive, systems‐level gene‐regulatory networks.^139^ In the context of neurodevelopment and histone lysine methylation, multi‐omics integration links epigenetic marks to gene regulation, cell fate, and developmental trajectories—merging disparate data into a coherent, mechanistic narrative to uncover the genetic programs that drive brain development.

## 7 TRANSLATIONAL PROSPECTS AND STRATEGIES

Building on the mechanistic insights above, developing interventions that target these processes has become a research hotspot. Current efforts center on small‐molecule drugs, metabolic interventions, and gene‐based technologies.

### 7.1 Small‐Molecule inhibitors and agonists

Because many NDDs arise from loss‐of‐function mutations in modifying enzymes, inhibiting the corresponding demethylases or boosting the relevant methyltransferases may offer a rational, “indirect” therapeutic approach by elevating overall methylation levels. For Kabuki syndrome—where KMT2D loss reduces H3K4me1—indirectly raising global H3K4 levels by inhibiting demethylases appears promising. Indeed, the KDM1A inhibitor TAK‐418 significantly increases global H3K4me1/2/3 in the brain and reverses disease phenotypes in models,^114^ suggesting KDM1A as a potential target. TAK‐418 is a novel small‐molecule compound that targets the catalytic FAD within the active site of LSD1 and irreversibly inhibits LSD1 activity. A human clinical trial showed that TAK‐418 exhibited a favorable safety profile and was well‐tolerated at daily doses up to 160 mg, with no dose‐limiting toxicities observed. TAK‐418 was rapidly absorbed and had a relatively short terminal elimination half‐life of 3.13–5.36 h. The results further indicated that TAK‐418 follows first‐order pharmacokinetics, with no evidence of saturation of clearance within the investigated dose range. After once‐daily administration for 10 consecutive days, pharmacokinetic parameters—including renal clearance—changed minimally; approximately 20% of TAK‐418 was excreted unchanged in urine within 24 h, while most of the drug was cleared from the circulation. TAK‐418 also reached peak concentrations rapidly in cerebrospinal fluid, suggesting ready penetration of the blood–brain barrier (BBB) and supporting its potential utility in patients with CNS disorders. Importantly, throughout the trial, there were no treatment discontinuations attributed to the drug, no serious adverse events, and no clinically meaningful changes in vital signs.^140^, ^141^ Beyond directly targeting “writers” and “erasers,” another route is to modulate reader proteins that recognize histone methyl marks. Designing small‐molecules that block repressive readers from binding chromatin, or that enhance the coupling of activating readers with transcription factors, could in principle fine‐tune specific gene programs with greater precision.^142^ However, this often brings about another issue that cannot be ignored, namely, the specificity and brain delivery efficiency of small‐molecule drugs. To improve efficiency, reduce systemic exposure, and achieve precise delivery, researchers are actively exploring brain‐targeted drug‐delivery strategies. Nanocarriers are nanoscale drug‐transport systems that enable targeted delivery and controlled release by encapsulating or conjugating active ingredients. Among them, Nanoemulgels represent a novel local delivery platform, formed by incorporating Nanoemulsions into a gel matrix, and are designed to address poor solubility of hydrophobic drugs, unpredictable absorption, and low oral bioavailability. This delivery approach offers high stability and ease of use, while avoiding gastrointestinal degradation and first‐pass metabolism. It can also achieve both immediate release and sustained delivery, and thus is receiving increasing attention for targeted therapy and improved safety.^143^ Prodrug design is a medicinal‐chemistry strategy in which a bioactive compound is chemically modified into a derivative that is inactive or less active in vitro; after reaching specific sites in the body, the derivative releases the parent active drug via enzymatic or non‐enzymatic reactions, thereby exerting pharmacological effects. This strategy is primarily used to optimize absorption, distribution, metabolism, and excretion properties, improve bioavailability, prolong duration of action, or reduce toxicity and side effects.^144^ Focused ultrasound combined with microbubbles is a physical, assisted delivery technology. After intravenous administration of microbubbles, focused ultrasound is applied to the target brain region to transiently and reversibly open the BBB, thereby allowing higher doses of drugs or nanocarriers to enter the brain. This technique has entered early clinical trials for treating brain tumors and neurodegenerative diseases, offering a potential avenue for future delivery of epigenetic therapeutics.^145^ Because systemic administration may cause off‐target effects and the BBB restricts drug entry into the brain, evaluating off‐target effects and specificity is a central component of safety assessment. The key question is whether, beyond precisely inhibiting the intended target protein, an inhibitor may inadvertently affect other proteins with similar structures or functions, thereby causing unexpected and potentially harmful biological effects. Five major dimensions of evaluation include: (i) assessing cross‐inhibition of other members within the same protein family; (ii) determining whether the inhibitor perturbs the homeostasis of other histone methylation marks; (iii) profiling unintended epigenetic and transcriptomic changes at the genome‐wide level in cellular or animal models; (iv) examining whether abnormal phenotypes or cytotoxicity occur in non‐target cell types or tissues; and (v) evaluating organ function and histopathological changes after systemic administration in animal models.

### 7.2 Metabolic intervention and nutritional modulation

Epigenetic modifications are strongly shaped by cellular metabolic state. S‐adenosylmethionine (SAM) is the methyl donor for histone methylation,^146^ therefore, dietary or supplement‐based manipulation of one‐carbon metabolism may indirectly influence histone methylation. Notably, a ketogenic diet improved hippocampal memory deficits in KMT2D‐deficient mice,^115^ potentially via the metabolite β‐hydroxybutyrate (BHB) acting as a histone deacetylase inhibitor or by altering methyl‐donor availability to impact the epigenome. Studies have shown that BHB is an endogenous and selective inhibitor of class I histone deacetylases (HDACs), and exogenous BHB administration increases global histone acetylation levels in mouse tissues. HDAC inhibition by BHB is associated with broad transcriptional changes, including altered expression of genes encoding oxidative stress–resistance factors such as forkhead box O3A (FOXO3A) and melatonin receptor 1B (MT2); BHB increases histone acetylation at the promoter regions of FOXO3A and MT2.^147^ Activated FOXO3A upregulates mitochondrial antioxidant enzymes, enhancing neuronal resistance to oxidative injury, which may confer protection in neurodegenerative diseases such as Parkinson's disease and Alzheimer's disease.^148^ FOXO3A also regulates energy metabolism and autophagy, thereby influencing the synthesis and clearance of synaptic proteins and participating in learning and memory processes.^149^ In regions such as the hippocampal dentate gyrus, FOXO3A modulates the proliferation and differentiation of neural stem/progenitor cells, affecting the generation of new neurons.^150^ MT2 cooperates with BDNF to exert neuroprotective effects and promotes neuronal survival, growth, and synaptic plasticity.^151^ In addition, BHB itself can serve as a substrate for the endogenous histone lysine β‐hydroxybutyrylation (Kbhb) modification, and the histone acetyltransferase p300 can catalyze increases in Kbhb,^152^ p300 also elevates H3K27ac levels, thereby recruiting histone methyltransferases and promoting increased H3K4me3. Neurological evidence further supports a positive role for BHB in the CNS. In hippocampal neurons, BHB enhances H3K4me3 levels at BDNF promoters I, II, IV, and VI, thereby promoting the expression of BDNF. Moreover, BHB treatment induces an overall increase in H3K4me3, an effect dependent on l‐type calcium channels. Activation of l‐type calcium channels by BHB triggers the Ca^2+^/CaMKII/CREB signaling pathway, promoting the binding of phosphorylated CREB (p‐CREB) and CBP to the BDNF promoter. These findings indicate that BHB regulates BDNF expression through coordinated modulation of cellular signaling and multiple histone modifications, revealing its broad regulatory roles in the CNS.^153^ Furthermore, in rats at postnatal Day 15, adding BHB to the culture medium not only allowed high‐frequency stimulation to effectively induce LTP of excitatory postsynaptic potentials (EPSPs), but also blocked EPSP–action potential facilitation, a phenomenon indicative of neuronal hyperexcitability. These results suggest that BHB can substitute for glucose as an energy substrate while protecting the structural integrity and stability of neurons, with particularly pronounced effects during early development.^154^ In addition, a ketogenic diet markedly reshapes metabolite pools in the liver and brain. Synthesis of the methyl donor SAM depends on the folate cycle and the methionine cycle, both of which are tightly coupled to the tricarboxylic acid cycle in energy metabolism. By reducing glucose utilization, a ketogenic diet may shift one‐carbon metabolic fluxes involving serine and glycine. Some studies have shown that ketogenic states upregulate the expression or activity of SAM‐synthesizing enzymes in the liver and brain, thereby increasing local SAM concentrations.^155^ This provides a more abundant substrate supply for histone methylation, including H3K4 methylation, and may partially compensate for reduced methylation efficiency caused by KMT2D mutations. Conversely, high‐fat diets (HFDs) (30%–50% fat over time) induce low‐grade systemic inflammation and can impair synaptic efficiency—possibly through HFD‐driven metabolic dysfunctions in synaptic protein synthesis and mitochondrial function that intersect with NDD‐related changes in plasticity.^156^ Additional dietary approaches—such as plant‐based diets,^157^ the Mediterranean diet,^158^ dietary antioxidants,^159^ and neuroprotective nootropic foods^160^—offer non‐pharmacologic avenues to ameliorate NDDs symptoms.

In summary, the core principle of the ketogenic diet is to induce a “ketogenic diet” by restricting carbohydrate intake. Under this condition, the body shifts to breaking down fats to generate ketone bodies as an alternative energy source. These ketone bodies are produced mainly in the liver and can be transported via the bloodstream to the brain and other tissues for utilization. Although the studies described above demonstrate that a ketogenic diet may improve cognitive function, it is not suitable for everyone. Populations for whom a ketogenic diet may be considered include individuals with cardiovascular disease, children with drug‐resistant epilepsy, patients with Parkinson's disease or Alzheimer's disease, individuals with obesity who require weight loss, those with type 2 diabetes or insulin resistance, and patients with polycystic ovary syndrome.^161^, ^162^, ^163^, ^164^, ^165^ By contrast, certain groups are not appropriate candidates for a ketogenic diet, such as individuals with metabolic abnormalities or chronic illnesses, pregnant or lactating women and adolescents, and those with eating disorders or sensitive gastrointestinal systems. For children who already have baseline feeding difficulties and growth retardation, the ketogenic diet can be particularly restrictive and difficult to implement. Long‐term adherence may also lead to a range of adverse effects, including headache, irritability, fatigue, constipation, an increased risk of kidney stones, electrolyte disturbances, dyslipidemia (especially elevated low‐density lipoprotein cholesterol), gastrointestinal discomfort, and vitamin and mineral deficiencies. Moreover, evidence remains limited regarding the long‐term impacts on growth and development, skeletal health, and cardiovascular outcomes.^166^, ^167^

## 8 CONCLUSION

Histone lysine methylation, particularly H3K4me3, plays a pivotal role in neurodevelopment and exhibits a clear “dose‐dependent” regulatory feature. At its core is the dynamic balance between H3K4 methylation “writers” and “erasers,” a balance that is essential for precisely controlling gene expression levels and ensuring the correct temporal and spatial patterns of neurodevelopmental events. The selective “erase” of one side of bivalent chromatin marks by demethylases—thereby resolving bivalent domains into a single active or repressive state—is a critical step toward differentiation into specific neuronal lineages. This process is not a simple “on/off” switch; rather, it depends on fine‐tuned regulation of H3K4me3 levels, and changes in its “dose” directly determine the extent and persistence of activation of lineage‐defining genes. Coordinated actions of methyltransferases and demethylases also ensure that the expression of migration‐related genes is maintained within a defined window; disruption of this balance perturbs the expression program of bivalent genes, leading to migratory defects and disordered differentiation timing, highlighting the importance of methylation homeostasis. Likewise, the expression of learning‐ and memory‐related genes is not all‐or‐none. Their transcriptional output depends on dynamic, opposing regulation of H3K4me3 at relevant loci by methyltransferases (e.g., the MLL family) and demethylases (e.g., the KDM5 family and LSD1). The balance between these enzymatic activities determines the “dose” of H3K4me3 accumulation at promoters, thereby fine‐tuning transcriptional strength and ultimately converting transient synaptic activity into long‐lasting, appropriately scaled changes in synaptic structure and function. In summary, normal neurodevelopment depends not merely on the presence or absence of H3K4me3, but more fundamentally on its precisely controlled “dose” This dose control is achieved through the cooperative and antagonistic interplay of methyltransferases and demethylases, and their balance constitutes a key epigenetic mechanism that ensures neurodevelopment proceeds at the right time, in the right place, and with the right intensity. In NDDs caused by loss of function of H3K4 methyltransferases, epigenetic dysregulation shows pronounced “cell‐type specificity.” This specificity determines that the core pathology of different diseases arises in particular cell populations and ultimately manifests as distinct clinical phenotypes. When abnormal H3K4 methylation occurs in NCSs or lineage‐restricted progenitors, it primarily affects fate determination, proliferation, and differentiation. For example, KMT2D plays an essential role in craniofacial neural crest cells; its mutation selectively disrupts enhancer H3K4me1 levels in this population, leading to dysregulated craniofacial and cardiac gene programs and thereby driving the characteristic facial dysmorphism and structural defects of Kabuki syndrome. Similarly, loss of KMT2D or SETD1A can impair neurogenesis in regions such as the hippocampus, disrupting the sustained production and proper integration of newborn neurons, which constitutes a cellular basis for the associated cognitive impairments. In contrast, when H3K4 methylation is disrupted in differentiated, mature neurons, the primary consequence is impairment of structural and functional maturation, especially synaptic plasticity and neural network integration. KMT2A is highly expressed in neurons of the prefrontal cortex and hippocampus; its mutation specifically leads to dendritic morphological abnormalities, reduced synaptic plasticity, and downregulation of memory‐consolidation gene programs in these higher cognitive regions, closely matching the severe intellectual disability and memory deficits observed in WSS patients. SETD1A is crucial in cortical projection neurons, and haploinsufficiency specifically disrupts axon guidance, dendritic complexity, and network synchronization in cortical neurons, which is directly linked to core schizophrenia symptoms such as working memory deficits and thought disorganization. Taken together, the role of H3K4 methylation regulation in NDDs is not uniform; rather, pathological outcomes depend strongly on the cell type and developmental stage at which dysregulation occurs. At the neural stem/progenitor stage, abnormal H3K4 methylation leads to lineage differentiation and neurogenesis defects; at the mature neuron stage, it leads to synaptic structural and plasticity abnormalities. This “cell‐type specificity” is key to understanding the heterogeneous clinical manifestations across syndromes and provides a theoretical basis for developing precision interventions targeting specific cellular populations. Finally, translational research on NDDs associated with abnormal H3K4 methylation is advancing along two prioritized directions: the development of safe, precise brain‐targeted small‐molecule therapies and the exploration of metabolic intervention and nutritional modulation.

## AUTHOR CONTRIBUTIONS

Yun Chen wrote the original draft. Yi Zhang, Hong Zhang, and Xiaoli Liang conceptualized this review and revised and edited the manuscript. All authors read and approved the final content of this manuscript.

## CONFLICT OF INTEREST STATEMENT

The authors declare no conflicts of interest.

## ETHICS STATEMENT

Not applicable.

## Data Availability

Data sharing is not applicable to this article as no datasets were generated or analyzed during the current study.
